# Prognostic value of the HALP score in breast cancer: a systematic review and meta-analysis

**DOI:** 10.3389/fonc.2025.1684940

**Published:** 2025-12-02

**Authors:** Jia Cai, Bo Wang, Niancai Jing, Yue Zhang

**Affiliations:** 1 College of Traditional Chinese Medicine, Changchun University of Chinese Medicine, Changchun, Jilin, China; 2 Department of Integrated Chinese and Western Medicine, Jilin Cancer Hospital, Changchun, Jilin, China

**Keywords:** breast cancer, HALP score, meta-analysis, systematic review, prognosis

## Abstract

**Background:**

The present research is a meta-analysis aimed at quantifying the influence of the hemoglobin, albumin, lymphocyte, and platelet (HALP) score on the overall survival (OS) and disease-free survival (DFS) in breast cancer patients. The objective of this research is to examine the prognostic significance of the HALP score in breast cancer patients and evaluate its predictive performance for survival outcomes.

**Methods:**

This research was conducted adhering to the Preferred Reporting Items for Systematic Reviews and Meta-Analyses (PRISMA) guidelines. We retrieved PubMed, EMBASE, Web of Science, Cochrane Library, and Google Scholar for studies relevant to the link between the HALP score and the prognosis of breast cancer up to July 3, 2025. The quality of the studies was appraised with Quality Assessment of Diagnostic Accuracy Studies-2 (QUADAS-2) and the Newcastle-Ottawa Scale (NOS). MetaDiSc 1.4, Stata SE 18, and Review Manager 5.4 were leveraged to examine the risk of bias and to conduct statistical analysis.

**Results:**

Among 971 studies, nine fulfilled the inclusion criteria, encompassing a total of 4,560 individuals. An increased HALP score was linked to favorable OS (HR = 0.61, 95% CI 0.39-0.97, p < 0.05). No statistically significant association was noted between the HALP score and DFS in breast cancer patients. The area under the summary receiver operating characteristics (SROC) curve (AUC) of the HALP score for predicting pathological complete response (pCR) was 0.57.

**Conclusion:**

Elevated HALP scores may represent a prognostic biomarker for favorable survival outcomes in breast cancer patients.

**Systematic review registration:**

https://www.crd.york.ac.uk/PROSPERO/myprospero identifier CRD420251031999.

## 1 Background

Breast cancer (BC) is a principal global health challenge, seriously jeopardizing women’s lives and health, and the incidence of BC is gradually increasing worldwide (1). However, substantial heterogeneity exists in the molecular expression profiles of BC. Factors such as the variations of gene expression patterns can influence the prognosis of BC (2). Despite advances in therapeutic approaches and medications, the overall survival (OS) of patients with BC remains suboptimal. Therefore, it is clinically meaningful to identify predictive factors that impact the progression and prognosis of BC. Currently, a number of immunonutritional biomarkers associated with BC have been identified, including peripheral neutrophil-to-lymphocyte ratio (NLR) (3) and prognostic nutritional index (PNI) (4).

Over recent years, the hemoglobin, albumin, lymphocyte, and platelet score (HALP) has emerged as a novel, cost-effective prognostic marker. It was initially proposed by Chen et al. in 2015 (5) to utilize the HALP score in forecasting the prognosis of gastric cancer. The score was computed as [hemoglobin (g/L) × albumin (g/L) × lymphocytes (/L)]/platelets (/L). Previously, a meta-analysis was executed to measure the influence of the HALP score on the prognosis of unselected solid tumors in 13,110 patients. The results denoted that a reduced HALP score was correlated with poorer OS (6). Some studies, however, suggested that no significant prognostic significance of the HALP score was discovered in BC (7).

Systemic inflammatory status and immune nutritional status are important indicators for assessing the prognosis of BC. The HALP score, as a comprehensive prognostic assessment tool, comprehensively reflects the patient’s systemic inflammatory level and immune nutritional status, providing a practical reference for predicting clinical prognosis and developing treatment strategies (8). It has been shown that the chronic inflammatory microenvironment can promote the occurrence and development of tumors through multiple mechanisms (9). In terms of lifestyle intervention, the study by Giosia et al. (10) shows that the Mediterranean diet pattern can significantly reduce the level of circulating inflammatory markers (such as TNF-α), which may be one of the potential mechanisms for reducing the risk of cancer.

BC-specific molecular subtypes are also key factors influencing patient prognosis. Previous studies have indicated that the progression of triple-negative BC (TNBC) is particularly closely related to the immune microenvironment (11). High levels of tumor-infiltrating lymphocytes (TILs) not only suggest that TNBC patients respond better to neoadjuvant chemotherapy but are also closely associated with improved survival outcomes. TNBC with significant TIL infiltration may be more sensitive to immunotherapy (12). The above evidence collectively suggests that the HALP score, as an integrative indicator reflecting systemic inflammatory status, may provide important references for predicting the prognosis of BC patients by assessing key pathophysiological processes related to tumor progression.

Therefore, the present research aims to quantify the prognostic utility of the HALP score for OS and DFS in BC patients via a meta-analysis. Besides, this research aims to assess whether the score can effectively enable early intervention in these patients and to better monitor and identify high-risk populations. In the meantime, the predictive performance of the score for pathological complete response (pCR) was also evaluated in individuals with BC.

## 2 Method

This study followed the Preferred Reporting Items for Systematic Reviews and Meta-Analyses (PRISMA) guidelines (13). The study protocol was registered in the PROSPERO database (CRD420251031999).

### 2.1 Search strategy

We retrieved related studies on PubMed, EMBASE, Web of Science, Cochrane Library, and Google Scholar through July 3, 2025. Search terms included “BC” and “HALP score”, as well as their combinations with Boolean operators (AND, OR, NOT). Search strategy details are delineated in **Supplementary Table S1**. Additionally, references of the included studies were manually screened to avoid omission.

### 2.2 Inclusion criteria and exclusion criteria

The inclusion criteria were comprised of (a) study design: observational studies, (b) population: BC patients, (c) with HALP scores measured as a categorical variable and comparative assessment between high and low scores, (d) with OS, DFS, and pCR as outcomes, and (e) no language restrictions. The exclusion criteria included (a) review articles, meta-analyses, or commentaries, (b) inadequate data for extraction, (c) animal or *in vitro* studies, (d) irrelevant research objectives, (e) non-BC populations, and (f) use of non-standard methods to calculate HALP.

### 2.3 Study selection

Two researchers (CJ and WB) independently screened the studies in EndNote, guided by the inclusion and exclusion criteria. Specifically, the titles and abstracts were reviewed and filtered, and then the full texts were downloaded and evaluated. In case of disagreement between the two researchers on the same study, it would be resolved by discussion with a third researcher (ZY).

### 2.4 Data extraction

Two researchers (CJ and WB) independently retrieved data and collected the following information with the predefined criteria: (a) study characteristics: first author, year of publication, type of study (cohort, case-control, among others), country, and sample size; (b) patients’ baseline information: age, gender, molecular typing, pathologic stage, Ki67 index, and lymph node metastasis; (c) treatment regimen: interventions and treatment strategies; (d) outcomes: HR and 95% CI for OS and DFS, and classification metrics of pCR, including true positives, false positives, true negatives, and false negatives. When the researchers held different opinions, it would be determined after consultation with a third researcher.

### 2.5 Quality assessment

Two researchers (CJ and WB) independently appraised the validity of the enrolled studies based on predefined criteria. Different evaluation tools were selected depending on the outcomes: cohort studies with OS as the outcome were appraised with the Newcastle-Ottawa Scale (NOS) (14). The scale encompassed three dimensions: selection, comparability, and outcome. The total score was 9 points. A score of 7–9 was considered high quality, 4–6 was considered medium quality, and less than 4 was considered low quality. Case-control studies were assessed using the corresponding version of NOS. The detailed information on the NOS scale is provided in **Supplementary Table S2**. Diagnostic studies with pCR as the outcome were appraised with Quality Assessment of Diagnostic Accuracy Studies-2 (QUADAS-2) (15). It examined the risk of bias and clinical applicability in four domains: patient selection, index test, reference standard, and flow and timing. The evaluation results were analyzed on Review Manager 5.4. All evaluation results were agreed upon through cross-checking, and if differences arose, they were resolved as above.

### 2.6 Statistical analysis

Stata SE 18 was leveraged for pooled analysis of the data from studies that reported OS and DFS outcomes, with HR and 95% CI as effect indicators. The I^2^ statistics were applied to quantify heterogeneity to determine whether to employ a random-effects model or a fixed-effects model. When the I^2^ statistic was > 50%, it denoted a high degree of heterogeneity, and thus a random-effects model was selected; otherwise, a fixed-effects model was applied. Subgroup analyses and regression analyses were executed by the countries of the study population, thus determining whether the country was a source of heterogeneity. A leave-one-out sensitivity analysis was executed to appraise the robustness of the findings. Egger’s test was employed to examine publication bias, with p < 0.05 denoting statistical significance. For studies reporting with pCR, MetaDisc 1.4 was leveraged to pool sensitivity, specificity, negative likelihood ratio (-LR), and positive likelihood ratio (+LR). The random-effects model was also employed to construct the summary receiver operating characteristic (SROC) curve and to compute the area under the curve (AUC) to investigate the diagnostic performance reported in the included studies.

## 3 Results

### 3.1 Study selection

**Figure 1:**
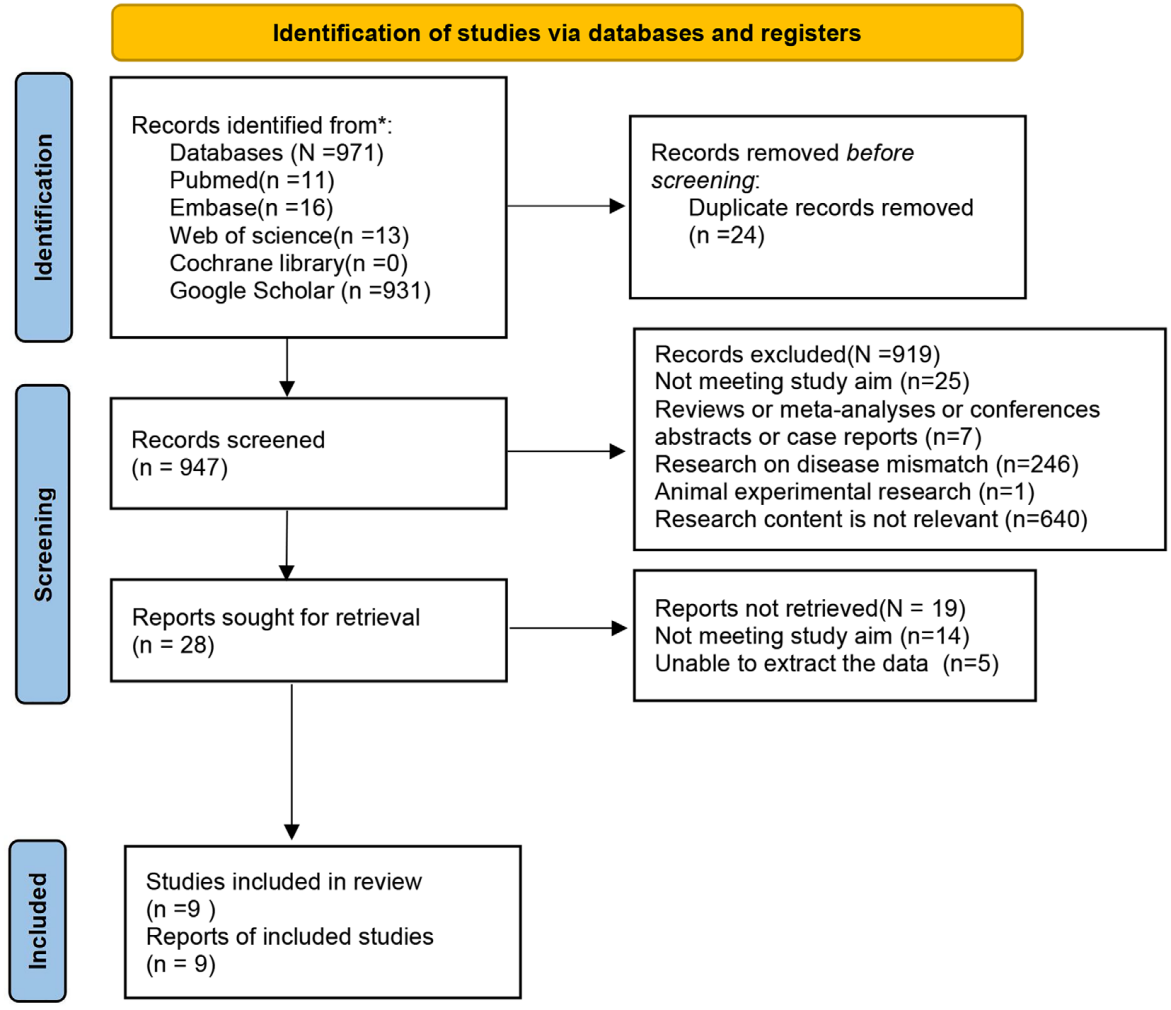
Flowchart of study search and selection.

In the present research, the database search yielded 971 studies. After deleting 24 duplicates, excluding 919 studies by the title and abstract, 19 by the full text, 9 studies were eventually included. The main reasons for exclusion included a mismatch with the scope or objective of the present study, inaccessible data, and a non-systematic review design. The detailed screening process is demonstrated in **Figure 1**.

### 3.2 Baseline characteristics

Nine studies were enrolled in this research (7, 16–23), comprising five from Turkey (7, 16, 18, 21, 23), three from China (17, 20, 22), and one derived from a United States database (19). In total, the research encompassed 4,560 patients. Three studies included male patients (7, 16, 18), amounting to nine male patients in total. Five studied female patients (17, 19–22), and one did not mention the gender of the participants. All studies were retrospective and were published in the last 5 years. Concerning molecular subtype, two studies included individuals with TNBC (20, 21). The outcome metrics in the included studies were different. Four studies reported on pCR (16–18, 23), two on OS (19, 20), one on OS and progression-free survival (PFS) (17), and two on OS and DFS (7, 21). The nine studies differed in the treatment regimens. One study enrolled those treated with surgery following neoadjuvant therapy (NAT) (17), one involved neoadjuvant chemotherapy (NAC) (18), two included post-NAC surgery (7, 22), one imposed no limitations on treatment regimen (21), one enrolled patients who were not surgical candidates and received NAC (predominantly the paclitaxel and carboplatin regimen), one enrolled patients who did not receive any preoperative chemotherapy (22), and one employed Doxorubicin/Cyclophosphamide → Docetaxel/Trastuzumab/Pertuzumab (AC-THP) combined with surgery. The details are delineated in **Table 1**.

**Table 1:** Baseline characteristics of the enrolled studies and patients.

Author	Publication Year	Country	Study design	Sample size	Age (yr)	Gender (n)	Treatment regimen	Molecular subtype (n)	Ki67 (n)	Stage (n)	Lymph node metastasis (n)	Outcomes
Jixin Fu (19)	2024	America	Retrospective cohort	639	NA	NA	NA	NA	NA	NA	NA	OS
Gözde Savaş (16)	2025	Turkey	Retrospective cohort	121	48.7581 ± 14.2557	F117M4	NAC+surgery	Hormone positive:80 Her2 positive:27 TNBC:14	≥50; 43 <50; 78	II:54 III:67	NO:3 lymph node-positive; 118	pCR
Shuqiang Liu (17)	2024	China	Retrospective cohort	789	51.8949 ± 9.4319	F789	NAT+surgery	HR(+)HER2(-); 369 HR(+)HER2(+); 111 HR(-)HER2(+); 134 HR(-)HER2(+); 175	≥30%; 434 15−30%; 213 <15%; 142	NA	N1+2:507 N3:282	pCR
Elif YUCE (18)	2023	Turkey	Retrospective cohort	127	50.3 ± 12.3	F125 M2	NAC	n=105 Luminal A; 23 Luminal B; 58 HER2 +; 14 Triple -; 10	n=76 ≤%10:22 >%10 ≤%20:23 >%20:31	NA	n=119 N+:99 N-:20	ORR;pCR
Caiyu Lou (20)	2022	China	Retrospective cohort	92	52.3 ± 8.9	F92	NAC and carboplatin as the main treatment drugs (no surgery indications)	TNBC	NA	II:32 III:60	N0 = 43 lymph node-positive:49	OS
Celal Alandag (21)	2022	Turkey	Retrospective cohort	166	50.3184 ± 10.8494	F166	No limitations	TNBC	≥50; 83 <50; 83	I; 23 II:87 III:53	NA	OS;DFS
Tongchao Jiang (22)	2024	China	Retrospective cohort	1856	48.7002 ± 11.8701	F1856	Adjuvant multimodal therapy after surgery	ER : Negative 546 Positive:1310 PR: Negative 695 Positive:1161 HER-2 status: Negative 1062 Positive:794	<14%:641 ≥14%:1215	I; 426 II:961 III:469	N0; 965 N1; 493 N2; 227 N3; 171	OS;PFS
SedatYildirim (7)	2024	Turkey	Retrospective cohort	624	>50 293 ≤50 331	F621 M3	NAC+surgery	Hormone positive and Her-2negative:293 Her-2 positive:244 Triple negative:87	≤20; 152 >20; 383 Missing; 89	NA	N0; 50 N1; 342 N2; 144 N3; 66 Missing; 22	OS;PFS
Mustafa Seyyar (23)	2025	Turkey	Retrospective cohort	146	52.3 ± 11.3	NA	AC-THP+surgery	HER2-Positive; 146 ER : Negative60 Positive86 PR: Negative70 Positive:76	NA	I:22 II:88 III:36	NA	pCR

NACT/NAC: neoadjuvant chemotherapy;NAT: neoadjuvant therapy; TNBC: triple-negative breast cancer; neoadjuvant chemotherapy; AC-THP: Doxorubicin＋Cyclophosphamide and Docetaxel+ Trastuzumab+ Pertuzuma;OS: Overall Survival;PFS: Progression-Free Survival; pCR: pathological Complete Response;DFS: Disease-Free Survival;ORR: Objective Response Rate.

### 3.3 Quality evaluation

**Figure 2:**
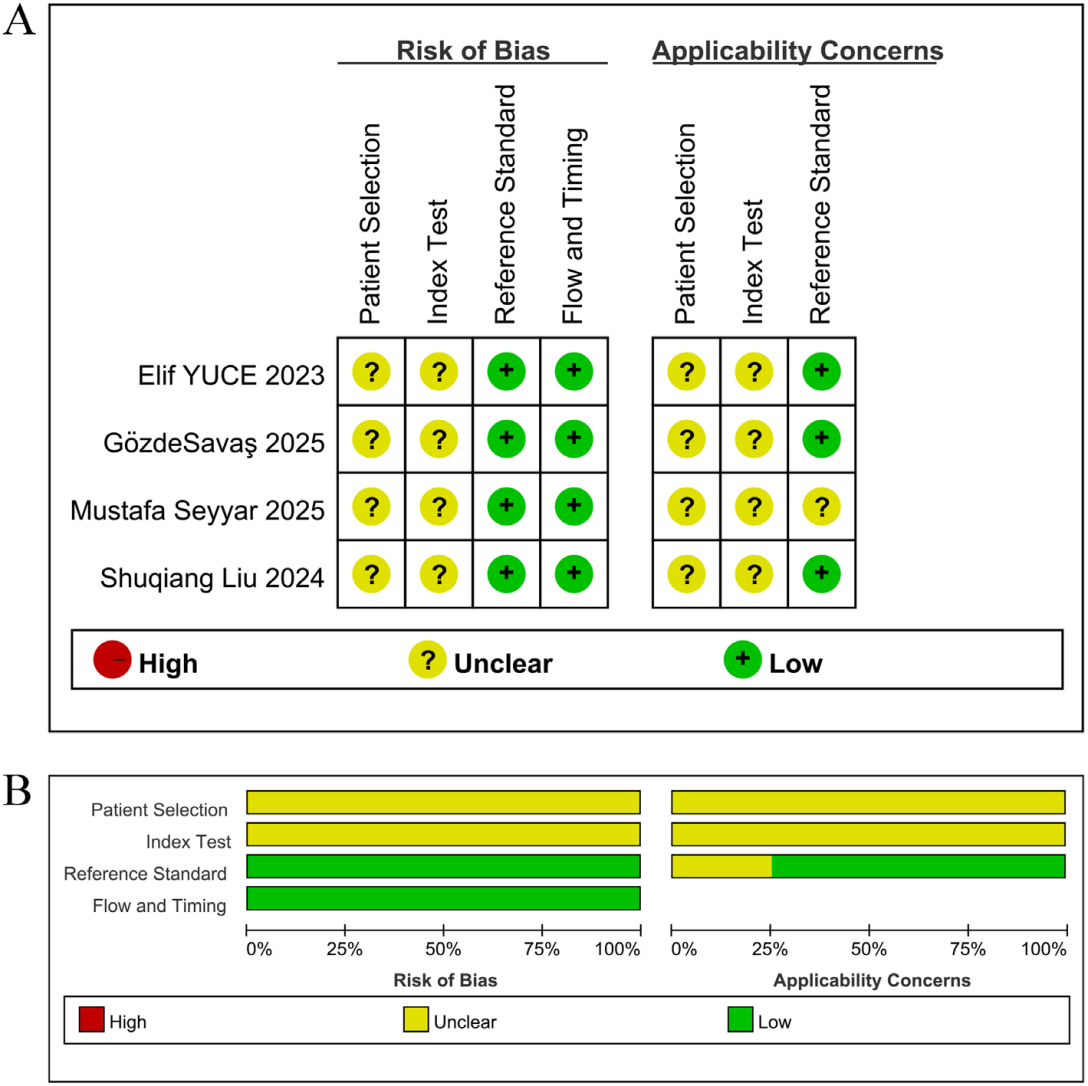
Risk of bias assessment, rated as low (+), medium ()? or high (-) risk per domain. **(A)** Risk of bias and applicability concerns for each included study; **(B)** Summary of the proportions of risk of bias and applicability concerns across all studies.

Among the nine included studies, five reported (7, 19–22) with OS as the primary outcome indicator, and thus were assessed with NOS. Studies scoring 7 and above on the scale were classified as high-quality, and the five studies all scored between 7 and 8 (mean score 7.5), as shown in **Table 2**. Four studies (16–18, 23) reported pCR, and thus QUADAS-2 was leveraged for quality assessment in these diagnostic studies. The results denoted that the included studies had high clinical applicability and a moderate risk of bias, as shown in **Figure 2**.

**Table 2:** Quality assessment with the Newcastle-Ottawa Scale for the studies with overall survival as an outcome.

Study	Selection				Comparability	Outcome			Quality scores
	Representativeness of the exposed cohort	Selection of the nonexposed cohort	Ascertainment of exposure	Demonstration that outcome of interest was not present at start of study	Comparability of cohorts on the basis of the design or analysis	Comparability of cohorts on the basis of the measurement	Was follow-up long enough for outcomes to occur	Adequacy of follow-up of cohorts	
Lou 2022 (20)	**☆**	**☆**	**☆**	**-**	**☆☆**	**☆**	**☆**	**☆**	8
Alandag 2022 (21)	**☆**	**☆**	**☆**	**-**	**☆☆**	**☆**	**☆**	**☆**	8
Jiang 2024 (22)	**☆**	**☆**	**☆**	**-**	**☆☆**	**☆**	**☆**	**☆**	8
Fu 2024 (19)	**☆**	**☆**	**-**	**-**	**☆☆**	**☆**	**☆**	**☆**	7
Yildirim 2024 (7)	**☆**	**☆**	**☆**	**-**	**☆☆**	**☆**	**☆**	**-**	7

### 3.4 The link of the HALP score with survival outcomes in BC patients

**Figure 3:**
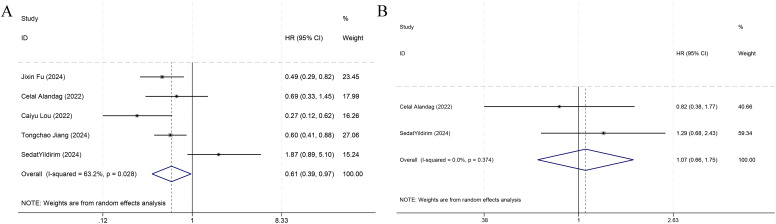
Forest plots demonstrating the link of HALP with overall survival **(A)** and disease-free survival **(B)**.

**Figure 4:**
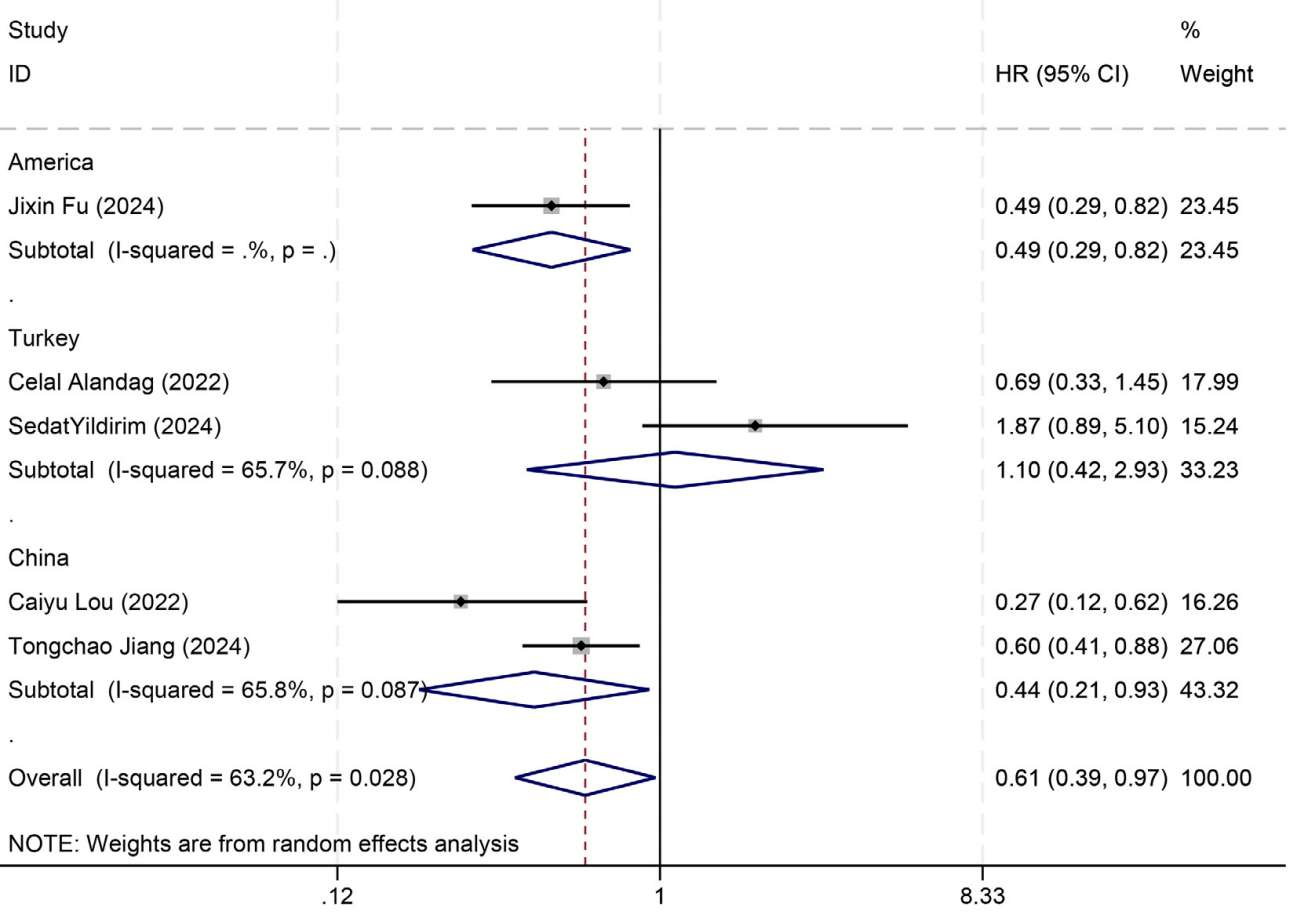
Subgroup analysis of HALP score and overall survival.

Among the nine included studies, five (7, 19–22) reported OS as the primary outcome, and thus a meta-analysis of the link of the HALP score with OS was implemented utilizing data from the five studies. The results denoted that individuals with an increased HALP score before treatment had notably longer OS (HR = 0.61, 95% CI 0.39-0.97, p < 0.05), but marked heterogeneity was observed between the studies (I² = 63.2%, p > 0.05) (**Figure 3**). Therefore, a subgroup analysis was executed on the effect of the country of the study population on OS (**Figure 4**). The analysis indicated that different countries were not the source of heterogeneity. Two of the studies reported HR values for DFS (7, 21) (pooled I² =0.0%, p=0.374), and thus a fixed-effects model was leveraged (HR = 1.07, 95% CI 0.66-1.75, p>0.05). The findings indicated no statistical significance (**Figure 3**).

The study of Jiang et al. (22) examined the prognostic utility of the HALP score for PFS. Via a multivariate Cox analysis, their study established that the HALP score was independently prognostic for PFS in BC patients (HR: 0.707, 95% CI 0.538-0.930, p=0.013). The study of Zhao et al. (24) examined the prognostic utility of the HALP score for recurrence-free survival (RFS) in 411 individuals with early invasive BC. The result denoted that reduced HALP scores demonstrated a significant correlation with poorer RFS (HR = 0.08, 95% CI 0.024-0.265, p < 0.0001).

**Figure 5:**
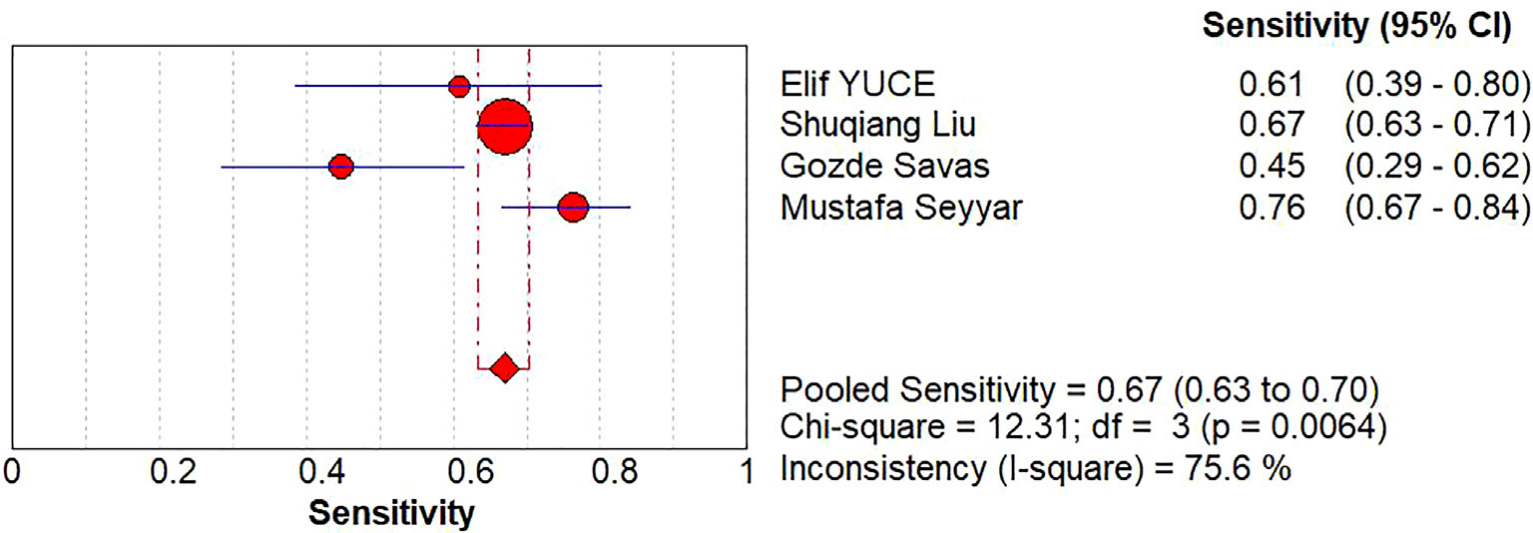
Sensitivity analysis of the HALP score for pathological complete response.

**Figure 6:**
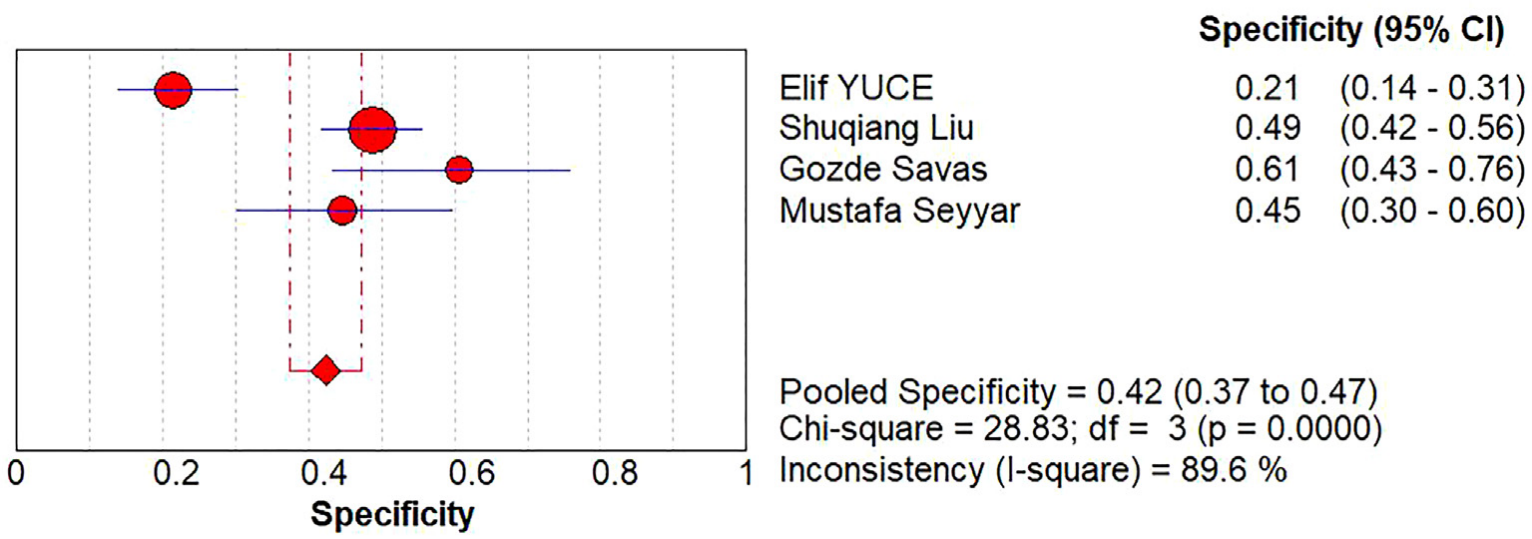
Heterogeneity analysis of the HALP score for pathological complete response.

**Figure 7:**
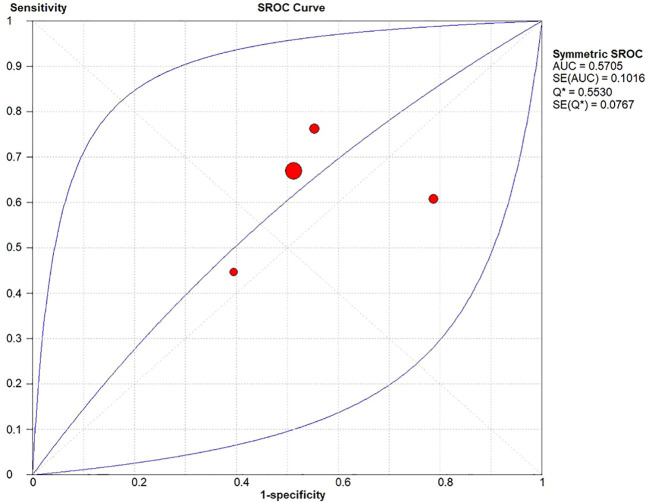
SROC curve of the HALP score for pathological complete response.

A diagnostic meta-analysis was executed on the four studies with pCR as the outcome (16–18, 23). The Spearman’s correlation coefficient was 0.4 (p=0.6>0.05), indicating no threshold effect, which allowed for a pooled analysis of sensitivity and specificity. The pooled sensitivity was 67% (95% CI: 63%-70%); specificity was 42% (95% CI: 37%-47%); +LR was 1.15 (95% CI: 0.90-1.47); -LR was 0.83 (95% CI: 0.56-1.22); and the diagnostic odds ratio (DOR) was 1.39 (95% CI: 0.73- 2.65). SROC curves were generated, and the AUC was 0.57. Forest plots of sensitivity and specificity are shown in **Figures 5**, **6**, and SROC curves are illustrated in **Figure 7**.

**Figure 8:**
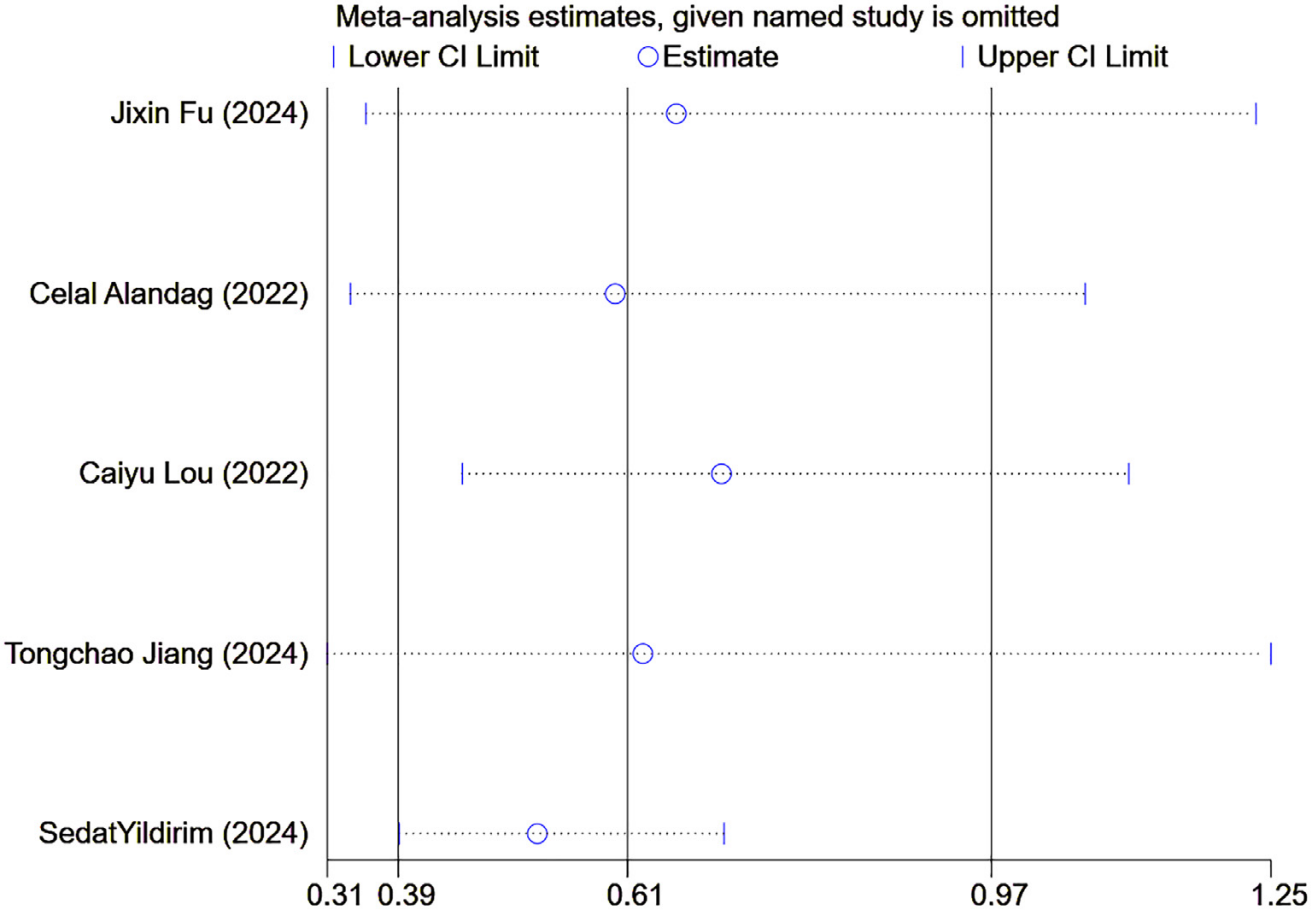
Sensitivity analysis of overall survival.

**Figure 9:**
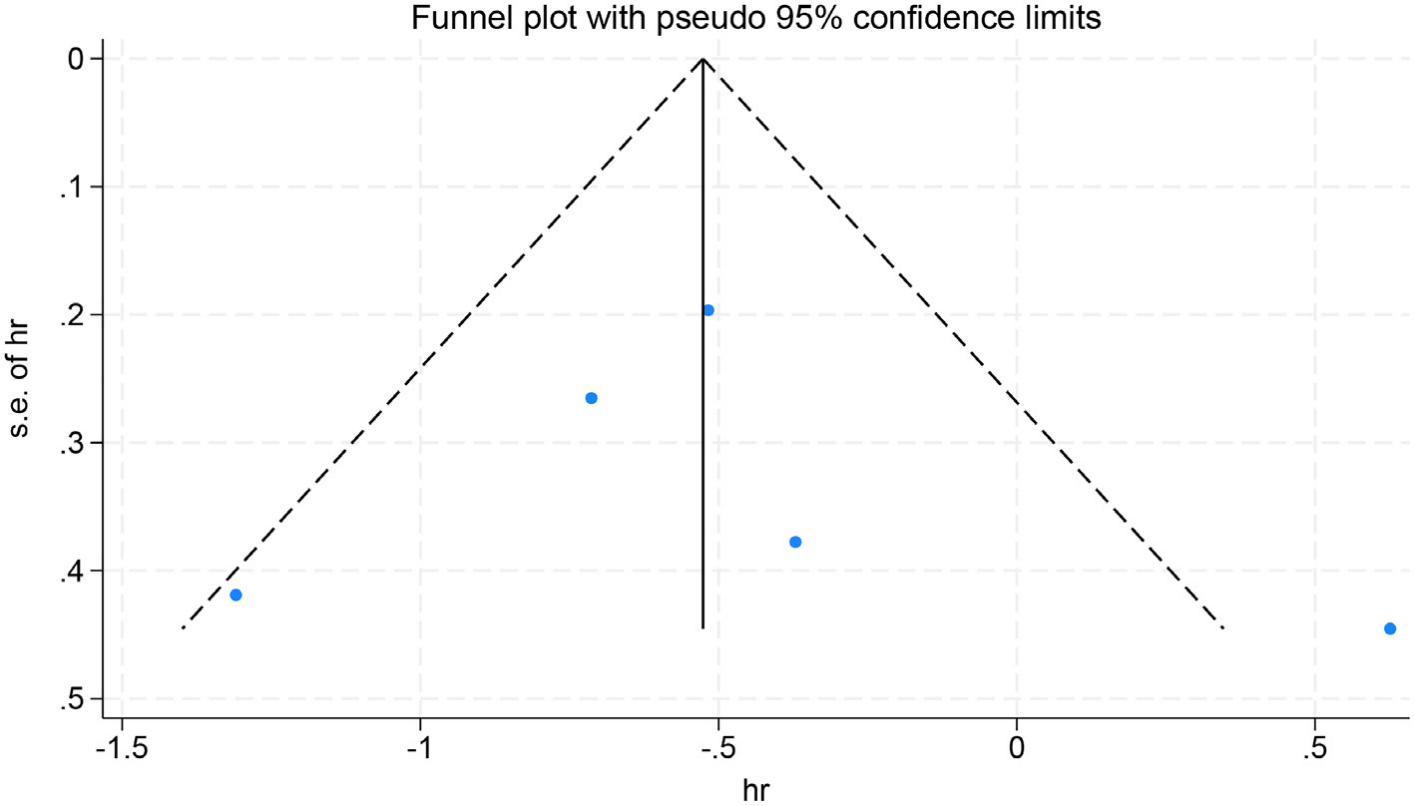
Publication bias analysis of overall survival.

Finally, sensitivity analyses and publication bias analyses were performed on the five studies with OS as the observed outcomes. The result of the sensitivity analyses remained robust when excluding any one of the five studies (**Figure 8**). No significant publication was detected in the Egger’s test (p=0.385), with symmetrical scatter points in the funnel plot, as illustrated in **Figure 9**.

## 4 Discussion

Accurate prediction of survival outcomes in BC patients is critical for delivering personalized therapy, and thus, investigating novel biomarkers to predict survival outcomes becomes imperative. Multiple contemporary studies have shown that a high HALP score is correlated with favorable survival outcomes in cancer patients. However, knowledge gaps persist in the prognostic utility of the HALP score in BC patients. Therefore, we performed a meta-analysis, including nine studies and 4560 BC patients, which preliminarily aimed to examine the prognostic and diagnostic performance of the HALP score in BC. We found that an elevated HALP score predicted improved OS in BC patients (p < 0.05), but lacked predictive value for DFS and demonstrated insufficient predictive power for pCR (AUC = 0.57).

In BC patients, the present study yielded consistent results for predicting OS, PFS, and RFS with previous studies (6, 25) for OS, PFS, and RFS, but found that the HALP score had no predictive value (p > 0.05) for DFS, which differed from prior findings (6, 25). Possible reasons are listed as follows: On the one hand, it may be explained by the paucity of studies on DFS (n=2), and the pooled effect estimates may be more susceptible to random variations or extreme values, reducing the stability of the results. On the other hand, the difference in results may also stem from the heterogeneity between studies, including different molecular types and treatment regimens. Even though the results were inconsistent with some studies, owing to the limited number of included reports, it is infeasible to adequately analyze these factors. This suggests that standardized, large-scale studies need to be conducted in the future to corroborate the prognostic capacity of the HALP score for DFS outcomes in BC. The diagnostic accuracy in the four studies with pCR as the outcome is limited. First, it may be owing to the disparate molecular subtypes of BC in the studies included. Studies have reported that HER2-positive (HR-negative) BC exhibits the highest rates of breast pCR (bpCR) and nodal pCR (npCR), and HER2-negative (HR-positive) BC exhibits the lowest rates of bpCR (26). Secondly, different definitions of pCR can also influence the results. Gözde Savaş et al. defined pCR as no evidence of invasive carcinoma and ductal carcinoma *in situ* (11), which is slightly different from the definitions of pCR in other studies. Finally, the different treatment regimens included in the studies can likewise affect the clinical and pathologic response and thus influence our results.

Hemoglobin, as a key molecule for oxygen transport, directly affects the blood’s oxygen-carrying capacity. When hemoglobin levels decrease, insufficient tissue oxygen supply can induce hypoxia in the tumor microenvironment, thereby activating hypoxia-inducible factors (such as HIF-1α), enhancing tumor invasion and metastasis, promoting angiogenesis, and ultimately driving malignant progression of BC and affecting patient prognosis (27). In addition, hemoglobin concentration also reflects the patient’s nutritional status, and its level is often used to define the degree of anemia in clinical practice. The trajectory of hemoglobin levels in the early stages of BC treatment is closely related to survival outcomes, while the mortality rate of the persistent anemia group is significantly higher than that of the normal hemoglobin group in multivariate analysis (28). It is worth noting that excessive secretion of TGF-β in the tumor microenvironment can inhibit erythrocyte production through organ-specific mechanisms, thereby leading to anemia (29). Multiple studies have confirmed that low hemoglobin levels before or during treatment are important predictors of poor disease control and reduced survival. Liang et al. (30) further demonstrate that in BC patients, those with low hemoglobin levels had a significantly increased risk of tumor metastasis.

Serum albumin is another important indicator of nutritional status, and a decrease in serum albumin level may indicate malnutrition or even cachexia. Albumin is able to reduce pro-inflammatory fatty acids, combat oxidation, and maintain plasma osmotic pressure. Therefore, higher albumin levels may improve patient survival by inhibiting tumor progression (31). Basic research has further shown that albumin can regulate the activity of autocrine growth factors, thereby affecting the proliferation of MCF-7 BC cells (32). Clinical data also confirm that low serum albumin levels are negatively correlated with the survival rate of patients with BC at all stages (HR = 3.53; P = 0.0033) (33).

In terms of immune regulation, lymphocytes, as a core immune component, induce tumor cell apoptosis through cytotoxicity. Reduced lymphocyte levels weaken immune surveillance and promote tumor progression (34). For example, in TNBC, for every 10% increase in TILs, patient survival increases by 10% (35). Therefore, lymphocyte-related inflammatory markers can provide important clues for the diagnosis, treatment, and prognostic assessment of BC (36).

Meanwhile, platelets play a complex role in tumor progression. Cancer patients often have a hypercoagulable state, which increases the risk of thromboembolism. More importantly, platelets directly participate in the regulation of tumor angiogenesis and metastasis by secreting a variety of chemokines and growth factors (such as VEGF and PDGF) (37–39).

Based on the above mechanisms, the HALP score, as an immunonutritional marker that comprehensively reflects a patient’s nutritional status, degree of anemia, and immune function, is closely related to the prognosis of BC (40). This score, by integrating multiple key indicators, provides an important basis for prognostic prediction, especially for BC patients generally with a high risk of malnutrition, and for older patients with a higher incidence of anemia, which has important clinical value (41, 42). There is a complex interaction between hemoglobin concentration and the tumor hypoxic microenvironment. On the one hand, tumor-induced oxygenation impairment can cause changes in blood rheology and increase blood viscosity. On the other hand, the decrease in hemoglobin caused by anemia may further aggravate tumor hypoxia, forming a vicious cycle (27, 43).

The HALP score represents a cost-efficient biomarker, which is dynamic and detectable. It can be repeatedly measured during treatment and rehabilitation to dynamically monitor changes in the patient’s physical condition. By integrating indicators of blood cell counts and albumin levels from routine blood tests, it can efficiently pinpoint BC patients with nutritional and immunological damage. This scoring system provides clinicians with an objective basis for assessment and helps to identify high-risk patients at an early stage. Thus, it assumes a critical role in optimizing the quality of life and extending survival duration for patients. Nevertheless, our findings revealed limited diagnostic validity of the score when using pCR as the outcome measure. Therefore, combined analysis with other biomarkers, such as NLR, should be performed in the future. The purpose is to improve the predictive performance for patient survival and prognosis and thus optimize clinical decision-making and individualized treatment.

There are several significant limitations of this study that warrant attention. First, with nine studies included, the sample size of the present research was relatively limited. This may reduce the statistical power of the current research and compromise the external validity of the findings. Second, all studies enrolled were retrospective in design. Despite the clinical feasibility of this approach, there is an unavoidable risk of selection bias and information bias, which makes it difficult to establish an exact causal relationship. Therefore, subsequent prospective multicenter studies need to be conducted for verification. In terms of population representativeness, the existing studies mainly focused on Asian populations (Turkish and Chinese patients) and did not examine populations in other regions, such as Europe and Africa. This limits the applicability of the findings on a global scale. Moreover, significant confounding bias may result from the disparities among studies in key variables such as treatment regimens, molecular types of BC (e.g., Luminal A, Luminal B, HER2-positive, TNBC) and HALP score cutoffs. This may trigger overestimation or underestimation of the effect sizes and increase inter-study heterogeneity. Notably, though the publication bias test showed no statistical significance, the limited number of included studies (n=5) may result in insufficient statistical power to fully exclude potential publication bias. Therefore, we recommend cautiously interpreting the pooled HR for OS. Future studies should focus on addressing these methodological limitations to offer a more reliable foundation for evidence-based medicine.

The present research validates that the HALP score demonstrates prognostic significance in BC, though its predictive utility for specific molecular subgroups remains unelucidated. Therefore, multicenter, large-sample, prospective studies need to be carried out in the future to substantiate our findings. The cutoff values for the HALP score varied across studies. Hence, they were standardized, and confounding bias was reduced to make the results more reliable. Current studies related to the HALP score are all retrospective in design and only obtain static data from patients at a single time point. Future studies should establish a dynamic monitoring system to achieve continuous tracking and evaluation of patients’ conditions. This will help reflect the clinical changes in patients more comprehensively and accurately.

## 5 Conclusion

The HALP score can efficiently forecast the OS of individuals with BC and has important value for prognostic assessment. Future prospective studies are required to validate this finding. Standardized assessment protocols are also needed to pinpoint the applicability of the HALP score in different subgroups of BC patients. Additionally, as HALP is an easily accessible prognostic indicator, the integration of HALP into existing risk prediction models may further enhance the predictive efficacy and is worth exploring in depth.

## Data Availability

The original contributions presented in the study are included in the article/**Supplementary Material**. Further inquiries can be directed to the corresponding author.
