# Supplementary material for: Prognostic value of the HALP score in breast cancer: a systematic review and meta-analysis
Source: Front Oncol. 2025 Dec 2;15:1684940. doi: 10.3389/fonc.2025.1684940 (PMC12705404; doi:10.3389/fonc.2025.1684940)
Supplement: Supplementary file 1 [file Table1.docx]

**Table S1** Search result records

| Pubmed | |
| --- | --- |
| # | Query |
| 1 | Breast Neoplasms[MeSH Terms] |
| 2 | "Breast Cancer"[Title/Abstract] OR "Breast Carcinoma*"[Title/Abstract] OR "breast malignanc*"[Title/Abstract] OR "Breast Neoplasm*"[Title/Abstract] OR "Breast Tumor*"[Title/Abstract] OR "Ca breast"[Title/Abstract] OR "cancer in the mammary gland"[Title/Abstract] OR "Cancer of Breast"[Title/Abstract] OR "cancer of the mammary gland"[Title/Abstract] OR "Human Mammary Carcinoma*"[Title/Abstract] OR "Human Mammary Neoplasm*"[Title/Abstract] OR "malignanc* of the breast"[Title/Abstract] OR "malignant breast neoplasm"[Title/Abstract] OR "malignant breast tumor"[Title/Abstract] OR "Malignant Neoplasm of Breast"[Title/Abstract] OR "Malignant Tumor of Breast"[Title/Abstract] OR "mamma* cancer*"[Title/Abstract] OR "mammary malignanc*"[Title/Abstract] |
| 3 | #1 OR #2 |
| 4 | "Hemoglobin, Albumin, Lymphocyte, and Platelet Score"[Title/Abstract] OR "HALP"[Title/Abstract] |
| 5 | #3 AND #4 |

| Embase | |
| --- | --- |
| # | Query |
| 1 | 'breast cancer'/exp |
| 2 | 'breast cancer':ti,ab,kw OR 'breast carcinoma*':ti,ab,kw OR 'breast malignanc*':ti,ab,kw OR 'breast neoplasm*':ti,ab,kw OR 'breast tumor*':ti,ab,kw OR 'ca breast':ti,ab,kw OR 'cancer in the mammary gland':ti,ab,kw OR 'cancer of breast':ti,ab,kw OR 'cancer of the mammary gland':ti,ab,kw OR 'human mammary carcinoma*':ti,ab,kw OR 'human mammary neoplasm*':ti,ab,kw OR 'malignanc* of the breast':ti,ab,kw OR 'malignant breast neoplasm':ti,ab,kw OR 'malignant breast tumor':ti,ab,kw OR 'malignant neoplasm of breast':ti,ab,kw OR 'malignant tumor of breast':ti,ab,kw OR 'mamma* cancer*':ti,ab,kw OR 'mammary malignanc*':ti,ab,kw |
| 3 | 'hemoglobin, albumin, lymphocyte, and platelet score':ti,ab,kw OR 'halp':ti,ab,kw |
| 4 | #1 OR #2 |
| 5 | #3OR #4 |

| Cochrane Library | |
| --- | --- |
| # | Query |
| 1 | MeSH descriptor: [Breast Neoplasms] explode all trees |
| 2 | (‘Breast Cancer’ OR ‘Breast Carcinoma*’ OR ‘breast malignanc*’ OR ‘Breast Neoplasm*’ OR ‘Breast Tumor*’ OR ‘Ca breast’ OR ‘cancer in the mammary gland’ OR ‘Cancer of Breast’ OR ‘cancer of the mammary gland’ OR ‘Human Mammary Carcinoma*’ OR ‘Human Mammary Neoplasm*’ OR ‘malignanc* of the breast’ OR ‘malignant breast neoplasm’ OR ‘malignant breast tumor’ OR ‘Malignant Neoplasm of Breast’ OR ‘Malignant Tumor of Breast’ OR ‘mamma* cancer*’ OR ‘mammary malignanc*’):ti,ab,kw |
| 3 | (‘Hemoglobin, Albumin, Lymphocyte, and Platelet Score’ OR ‘HALP’):ti,ab,kw |
| 4 | (#1 OR #2) AND #3 |
| 5 |  |

| Web of Science | |
| --- | --- |
| # | Query |
| 1 | TS=((Breast Cancer) OR (Breast Carcinoma*) OR (breast malignanc*) OR (Breast Neoplasm*) OR (Breast Tumor*) OR (Ca breast) OR (cancer in the mammary gland) OR (Cancer of Breast) OR (cancer of the mammary gland) OR (Human Mammary Carcinoma*) OR (Human Mammary Neoplasm*) OR (malignanc* of the breast) OR (malignant breast neoplasm) OR (malignant breast tumor) OR (Malignant Neoplasm of Breast) OR (Malignant Tumor of Breast) OR (mamma* cancer*) OR (mammary malignanc*)) |
| 2 | TS=((Hemoglobin, Albumin, Lymphocyte, and Platelet Score) OR (HALP)) |
| 3 | #1 AND #2 |

| Google Scholar | |
| --- | --- |
| # | Query |
| 1 | TS=((Breast Cancer) OR (Breast Carcinoma*) OR (breast malignanc*) OR (Breast Neoplasm*) OR (Breast Tumor*) OR (Ca breast) OR (cancer in the mammary gland) OR (Cancer of Breast) OR (cancer of the mammary gland) OR (Human Mammary Carcinoma*) OR (Human Mammary Neoplasm*) OR (malignanc* of the breast) OR (malignant breast neoplasm) OR (malignant breast tumor) OR (Malignant Neoplasm of Breast) OR (Malignant Tumor of Breast) OR (mamma* cancer*) OR (mammary malignanc*)) |
| 2 | TS=((Hemoglobin, Albumin, Lymphocyte, and Platelet Score) OR (HALP)) |
| 3 | #1 AND #2 |
