# Supplementary material for: Prognostic value of the HALP score in breast cancer: a systematic review and meta-analysis
Source: Front Oncol. 2025 Dec 2;15:1684940. doi: 10.3389/fonc.2025.1684940 (PMC12705404; doi:10.3389/fonc.2025.1684940)
Supplement: Supplementary file 2 [file Table2.docx]

**Table S2 Quality assessment criteria of the Newcastle-Ottawa Scale (NOS) for cohort studies**

**I. Cohort Selection (Maximum 4 stars )**

1. **Representativeness of the exposed cohort**
   - **【Scoring Criteria】**
     - **Award a star (☆):** One of the following conditions must be met:
       - a. The study cohort is drawn sequentially or randomly from a well-defined, representative breast cancer population (e.g., all newly diagnosed breast cancer patients within a specific time period from a regional or national cancer registry).
       - b. Although not a strictly random sample, the source population is well representative (e.g., all breast cancer patients treated at a large general hospital during a specific period, and the hospital is the main treatment center in the area).
     - **No stars:**
       - c. Poor cohort representativeness, such as selecting only young patients from a particular hospital, patients with specific molecular subtypes, or volunteers, which can lead to significant selection bias.
       - d. The study does not describe the source of the research subjects.
2. **Selection of non-exposed queues**
   - **【Scoring Criteria】**
     - **Award a star(☆):**
       - a. **The high HALP score group** must be from the same breast cancer patient population **at the same time and institution as the low HALP score group** . That is, all patients come from the same source population.
     - **No stars:**
       - b. From different source populations (e.g., patients selected from another hospital as controls).
       - c. The source of the high HALP score group is not described.
3. **Determination of exposure**
   - **【Scoring Criteria】**
     - **Award a star (☆):** The following conditions must be met:
       - a. **The HALP score** is calculated based on objective and accurate medical records (such as laboratory test results in electronic medical records: hemoglobin, albumin, lymphocytes, platelets) and obtained through a predetermined formula. This is the ideal situation.
       - b. The study had a clear and standardized protocol for collecting blood samples and calculating scores, ensuring **consistency in the determination of exposure for all patients** .
     - **No stars:**
       - c/d. The calculation method for the HALP score is unclear, or there are a large number of missing data and no explanation is given on how to handle them.
4. **At the start of the study, no participants had already experienced the outcome of interest.**
   - **【Scoring Criteria】**
     - **Award a star (☆):**
       - The study elucidated that all participants **did not develop** the studied prognostic outcome (e.g., no death, recurrence, or metastasis) at the time of enrollment (i.e., when the baseline HALP score was determined). This is a fundamental requirement for cohort studies.
     - **No stars:**
       - b. The study included patients who had already experienced an outcome at the start of the study (e.g., patients in the terminal stage were included) .

**II. Comparability (maximum 2 stars )**

1. **Comparability of exposed** **cohort and non-exposed cohort**
   - **【Scoring Criteria】**
     - **Award a star (maximum 2 stars ):** This item is to assess whether the study has controlled for confounding factors in the analysis. 1 star is awarded if significant confounding factors are properly controlled , up to a maximum of 2 stars.
     - **First star (☆): The study must** have statistically adjusted for **the most important factors that are strongly correlated with HALP scores and breast cancer prognosis** in the analysis. **Core factors typically include:**
       - **Tumor staging**
       - **age**
     - **Second star (☆):** In addition to the factors mentioned above, **other important prognostic factors** are adjusted. **Common factors include:**
       - **Tumor grading**
       - **Molecular typing (e.g., ER, PR, HER2 status)**
       - **The treatment options (surgery, chemotherapy, radiotherapy, endocrine therapy, etc.)**
       - **Comorbidities**

**III. Results (Maximum 3 stars )**

1. **Result determination method**
   - **【Scoring Criteria】**
     - **Award a star (☆):** One of the following conditions must be met:
       - a. The determination of the outcome event (such as death or relapse) is made independently by a committee or physician **who is unaware of the patient's HALP score grouping (blinded method).** This is the most rigorous method.
       - b. The determination of the outcome depends on **objective, reliable and unbiased records**, such as links to official **population mortality registries** and standardized imaging assessment reports of tumor recurrence.
     - **No stars:**
       - Determining outcomes based on unverified self-reported records is prone to misclassification bias.
2. **Is the follow-up period long enough?**
   - **【Scoring Criteria】**
     - **Award a star (☆):**
       - a. The study report should state a median follow-up time that is **reasonable** and sufficient to observe a adequate number of prognostic events (such as death or relapse). Five years or longer is preferred. It is also necessary to report that a sufficient number of events actually occurred during this period (e.g., total number of deaths > 50), rather than the vast majority of patients failing to reach the endpoint.
     - **No stars:**
       - b. The follow-up period is too short, and the vast majority of patients have not yet experienced the outcome event, making it impossible to draw meaningful prognostic conclusions.
3. **Follow-up adequacy**
   - **【Scoring Criteria】**
     - **Award a star (☆):** One of the following conditions must be met:
       - **100% complete follow-up** was achieved, and all enrolled patients had a clear outcome status.
       - b. **The loss to follow-up rate is low** (generally **< 25 % is** considered to be acceptable; the lower, the better), and the study **clearly reports the loss to follow-up rate**. At the same time, **a comparative analysis of the baseline characteristics of those who lost to follow-up and those who completed follow-up is conducted** to prove that there were no significant differences in key characteristics between the two groups. Therefore, the bias caused by loss to follow-up is small.
     - **No stars:**
       - c. The loss to follow-up rate is high (e.g., >25 %), and the individuals lost to follow-up have not been described or analyzed.
       - d. No information is provided regarding loss to follow-up.

**Total score and quality grade classification**

- **Total score calculation: Add up the asterisks (**☆) obtained from the above 8 items .
- **Quality grade:**
  - **High quality:** 7 - 9 stars
  - **Medium quality:** 4 - 6 stars
  - **Low quality:** 0 - 3 stars
